# Clinical significance of CYP11B2 immunostaining in unilateral primary aldosteronism

**DOI:** 10.1530/EC-23-0344

**Published:** 2024-01-12

**Authors:** Marianna Viukari, Helena Leijon, Tiina Vesterinen, Sanni Söderlund, Päivi Hämäläinen, Iina Yliaska, Päivi Rautiainen, Reeta Rintamäki, Minna Soinio, Ilkka Pörsti, Pasi I Nevalainen, Niina Matikainen

**Affiliations:** 1 Endocrinology, Helsinki University Hospital and University of Helsinki, Helsinki, Finland; 2 Department of Pathology, University of Helsinki and HUS Diagnostic Center, Helsinki University Hospital, Helsinki, Finland; 3 Department of Internal Medicine, Tampere University Hospital, Tampere, Finland; 4 Medical Research Center Oulu, Oulu University Hospital and Research Unit of Internal Medicine, University of Oulu, Oulu, Finland; 5 Joint Municipal Authority for North Karelia Social and Health Services (Siun Sote), Joensuu, Finland; 6 Department of Endocrinology and Clinical Nutrition, Kuopio University Hospital, Kuopio, Finland; 7 Department of Endocrinology, Turku University Hospital, Turku, Finland; 8 Faculty of Medicine and Health Technology, Tampere University, Tampere, Finland

**Keywords:** adrenal venous sampling, functional subtyping, primary aldosteronism, CYP11B2 immunostaining, adrenalectomy outcome

## Abstract

**Objective:**

The associations between adrenal histopathology, lateralization studies, and surgical outcomes in primary aldosteronism remain poorly characterized. We examined the value of immunohistochemical analysis of CYP11B2 for evaluation of adrenalectomy outcomes after anatomical versus functional subtyping.

**Design:**

A retrospective multicenter study of 277 patients operated for primary aldosteronism who had an adrenalectomy sample available in the Finnish biobanks from 1 January 2000 to 31 December 2019. Adrenal slides from biobanks were analyzed centrally after CYP11B2 and CYP11B1 staining. Clinical data were obtained from patient registries. Histopathological diagnosis and cure after surgery were assessed as outcome measures.

**Results:**

Re-evaluation with CYP11B2 staining changed the histopathological diagnosis in 91 patients (33%). The presence of a CYP11B2-positive adenoma and the use of functional subtyping independently predicted clinical cure of primary aldosteronism. CYP11B2-positive <7 mm nodules were more frequent in patients without clinical cure, whereas CYP11B2-positive micronodules were common in all patients and had no impact on adrenalectomy outcomes. Small CYP11B2-positive nodules and micronodules were equally prevalent regardless of the subtyping method applied. Clinical cure rates were lower and CYP11B2-negative adenomas more common after adrenalectomy based on anatomical imaging than functional studies.

**Conclusions:**

Incorporating CYP11B2 staining in histopathological diagnosis enhances the prediction of surgical outcomes in primary aldosteronism. A finding of CYP11B2-positive adenoma is indicative of cure of primary aldosteronism, whereas smaller CYP11B2-positive nodules associate with poorer results at postoperative evaluation. Functional subtyping methods decrease the operations of CYP11B2-negative adenomas and are superior to anatomical imaging in identifying unilateral primary aldosteronism.

## Introduction

Primary aldosteronism (PA) is the most common form of secondary endocrine hypertension and predisposes to increased cardiovascular morbidity and mortality partly independent of hypertension stage (1, 2, 3). Although targeted treatment is essential in PA, this requires prompt recognition of unilateral versus bilateral PA subtypes. In unilateral disease, surgical treatment is associated with higher rates of controlled hypertension, reduced rates of atrial fibrillation and chronic kidney disease, improved quality of life, and lower all-cause mortality when compared with medical therapy with mineralocorticoid receptor antagonists (MRA) (4, 5, 6, 7).

The significance of adrenal histopathological examination in the diagnostic workup of PA has been emphasized by the recent International Histopathology Consensus for Unilateral Primary Aldosteronism (HISTALDO) (8). As hematoxylin–eosin (H&E) staining alone does not reveal the site of aldosterone production, this consensus recommends that the histopathological diagnosis of PA should be based on morphology using H&E staining plus immunohistochemistry using CYP11B2 staining to verify the presence of autonomous aldosterone secretion (8, 9). HISTALDO unified the nomenclature to aldosterone-producing adenomas (APAs), aldosterone-producing nodules (APNs), aldosterone-producing micronodules (APMs), or aldosterone-producing diffuse hyperplasia (APDH) (8). However, mixed findings and unilateral cases with nonclassical histopathology due to APN and APM exist (10, 11, 12). Previous studies, with sample sizes ranging 60–98 subjects, have shown that in unilateral PA, patients with nonclassical adrenal histopathology (i.e. the absence of a CYP11B2-positive adenoma) exhibit a higher incidence of postsurgical disease and increased aldosterone production from the remaining adrenal (8, 10, 12, 13). This indicates the need for follow-up even when initial adrenalectomy results imply a cure.

The gold standard for preoperative PA subtyping, adrenal venous sampling (AVS), is technically challenging, has limited availability, and no definitive decision-making cutoffs have been established (1, 14, 15). Consequently, there is an unmet need for less demanding functional imaging for lateralization diagnostics. The previously applied [^131^I]norcholesterol (NP-59) scintigraphy has been abandoned due to limited sensitivity (16, 17). Novel tracers specific for CYP11B2 are being developed, as ^11^C-metomidate positron emission tomography (^11^C-MTO-PET), currently in clinical use in some centers, does not optimally detect unilateral PA in the absence of an adrenal nodule in anatomical imaging (14, 18, 19, 20).

The superiority of any lateralization method has been difficult to determine partly because of a lack of final immunohistochemical diagnosis. The only existing prospective study comparing CT and AVS in PA subtyping did not reveal benefit in achieving postoperative biochemical or clinical cure with AVS (21), although retrospective studies have shown better adrenalectomy results after AVS (22, 23). Patients with nonclassical adrenal histopathology in CYP11B2 staining have been shown to exhibit increased ratio of absolute aldosterone concentration in the contralateral adrenal vein to peripheral vein at AVS (10). Clearly, modern immunohistochemistry reveals more complex subtypes than classical APA or bilateral hyperplasia, but their relationship to cure rates or preoperative lateralization results remains largely unknown.

In the present study, we evaluated the outcomes of a retrospective cohort of patients who had undergone unilateral adrenalectomy due to PA during 2000–2019 and had biobanked adrenal tissue available for reassessment of histopathological diagnosis. Here, we reclassified the samples to APA, APN, APM, APDH and to CYP11B2-negative adenomas. Our aims were to study (i) how immunohistochemical analyses of CYP11B2 and CYP11B1 impact the histopathological diagnosis; (ii) the significance of histopathological findings for the adrenalectomy outcomes; and (iii) whether CYP11B2 and CYP11B1 staining positivity is associated with lateralization in anatomical imaging or functional methods including AVS, ^11^C-MTO-PET, and adrenal scintigraphy.

## Patients and methods

We identified all patients with clinically verified PA (ICD-10 code E26.0) who were operated between 1 January 2000 and 31 December 2019 and had adrenal tissue in the Finnish biobanks. The biobanks providing histological adrenalectomy samples were the Helsinki Biobank, Biobank Borealis of Northern Finland, Biobank of Eastern Finland, Finnish Clinical Biobank Tampere, and Auria Biobank. In addition, samples were retrieved from the Siun Sote hospital district.

The study was approved by the Ethics Committee of Helsinki University Hospital (HUS 1352/2018).

We obtained samples from 255 patients; all patients fulfilled the criteria for clinically verified primary hyperaldosteronism (E26.0) (14). Four samples were excluded from the study, one due to the finding of adrenocortical carcinoma, two due to nonrepresentative samples, and one due to the patient not having undergone adrenalectomy but postmortem obduction. In addition, 22 samples from a previous prospective clinical study were included (18).

Clinical and biochemical patient data were collected from local hospital records, including radiology and nuclear medicine reports and AVS results. Antihypertensive medication was reported as daily defined doses (DDD).

### Histopathology

Histopathological reexamination, including staining with H&E, CYP11B2, and CYP11B1 was performed centrally in Helsinki University Hospital by a single pathologist with special expertise in endocrine pathology (HL). The methods have been described previously (15, 18). The APA group included cases of PA with a CYP11B2-positive nodule ≥7 mm, whereas the non-APA group included samples with no APA present. Nonclassical histopathological features, including APNs (in this study defined as round CYP11B2-positive nodules <7 mm), APMs, and APDH were recorded for both the APA and the non-APA groups. CYP11B2 and CYP11B1 scores were calculated by multiplying the staining intensity (scale 0–3) by the percentage of stained cells. CYP11B2 scores were only calculated for APAs. Original histopathological reports were collected from local hospital records with patients classified as having adrenal adenoma or hyperplasia according to H&E staining by local pathologists.

Histopathological diagnosis was considered changed if the adenoma in H&E staining lacked CYP11B2 positivity by the reevaluation or a CYP11B2-positive APA was found in a sample that had been classified as hyperplasia according to H&E staining in the original histopathological report.

### Anatomical imaging of the adrenals

Radiology reports for adrenal CT and MRI performed according to local protocols were collected.

### Adrenal venous sampling

All AVS studies were performed in Tampere University Hospital. Stimulated AVS was routinely performed during continuous 50 µg/h cosyntropin infusion. Catheterization was considered successful when the selectivity index (SI = AV/inferior vena cava (IVC) cortisol concentrations) on both sides is ≥5. PA was classified as unilateral based on a lateralization index (LI = aldosterone–cortisol ratio between the dominant and contralateral AVs) ≥4. Contralateral suppression index (CSI = aldosterone–cortisol ratio between the nondominant AV and IVC) values <1 suggested unilateral disease when LI was between >3 and <4.

### Functional imaging with radioisotopes

All ^11^C-MTO-PET studies were performed in Turku PET center without dexamethasone pretreatment as previously described (18). The scans were analyzed by an expert in nuclear medicine, although standardized uptake values were inaccessible from biobank data.

Adrenal scintigraphy studies were performed during the entire study period in local centers and evaluated by an expert in nuclear medicine.

### Evaluation of surgical outcome

Clinical outcomes for each subject were determined according to the Primary Aldosteronism Surgical Outcome (PASO) criteria (24). Biochemical cure was classified as either present or absent due to the small number or postoperative renin and aldosterone measurements. See Supplemental methods for further information.

### Statistical analysis

Results are expressed as either number and percentage, mean and standard deviation, or median and interquartile range, as appropriate. Comparisons of independent samples were performed with independent-samples *t*-test, Mann–Whitney *U*-test, or Fisher’s exact test as appropriate. Associations between continuous variables were examined using Pearson’s or Spearman’s rank correlation coefficient. Binary logistic regression was performed to determine independent predictors of achieving clinical cure. IBM SPSS Statistics version 28.0.0 was used for analysis.

## Results

### Histopathology

**Figure 1:**
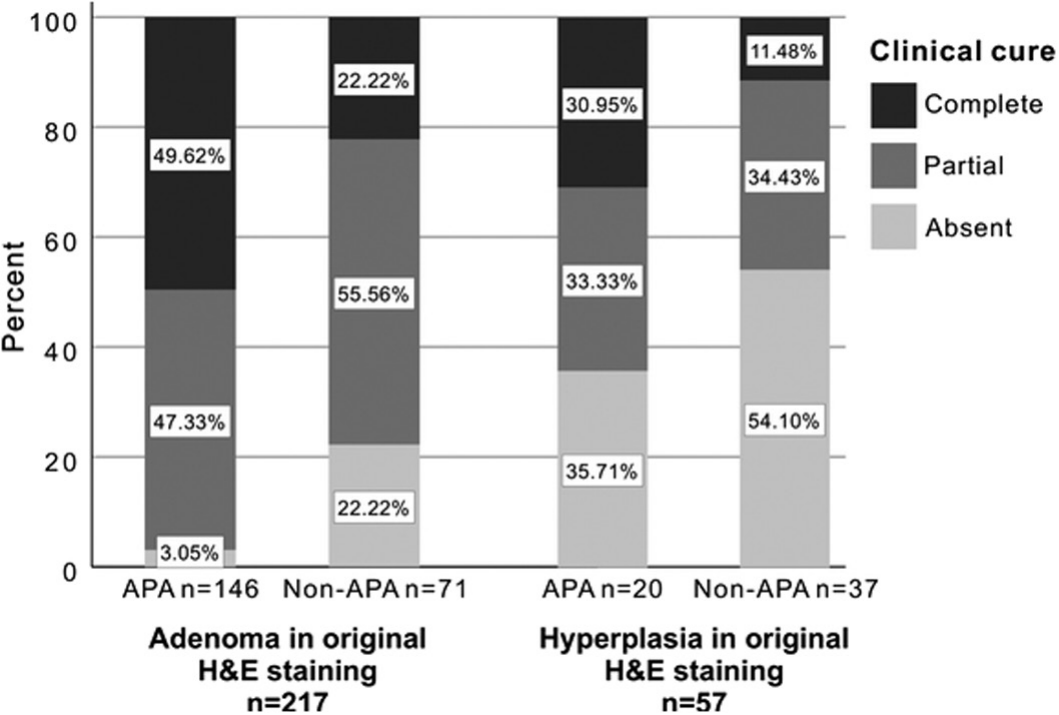
Rate of clinical cure according to adrenal histopathology evaluated by hematoxylin–eosin and CYP11B2 staining. APA, aldosterone-producing adenoma; H&E, hematoxylin–eosin.

Information on the original histopathological diagnosis according to H&E staining was available in 274 cases (Fig. 1). Reevaluation with CYP11B2 staining changed the histopathological diagnosis in 91 patients (33.2%). Table 1 presents the baseline characteristics of all 277 patients, and according to the division of APA and non-APA groups based on CYP11B2 staining. Those with non-APA (39.7%) were more often male, older and had lower aldosterone–renin ratio (ARR), lower 24-h aldosterone excretion, and higher plasma potassium than those with APA (60.3%). Additionally, non-APA patients had higher frequency of cardiovascular disease and higher plasma creatinine. Hypertension severity was similar between the groups.

**Table 1:** Preoperative patient characteristics of patients with APA and non-APA according to histopathological classification.

Variable (reference range, unit)	Data available, *n*	All patients, *n* = 277	APA, *n* = 167	Non-APA, *n* = 110
Sex, *n* (female/male)	277	139/138	99/68	40/70^b^
Age, years	277	52.1 ± 11.5	49.1 ± 11.5	56.6 ± 9.9^b^
BMI, kg/m²	250	29.5 ± 5.4	28.9 ± 5.7	30.3 ± 4.9
Hypertension duration, years	215	7.0 (2.0–14.0)	7.0 (2.0–11.0)	10.5 (2.5–19.8)^a^
Cardiovascular disease, *n* (%)	273	24 (8.8%)	7 (4.2%)	17 (15.6%)^a^
Diabetes, *n* (%)	277	50 (18.1%)	24 (14.4 %)	26 (23.8%)
Systolic BP, mm Hg	240	152 (140–170)	153 (140–170)	152 (139–170)
Diastolic BP, mm Hg	240	93 (85–100)	93 (85–100)	91 (84–100)
Serum aldosterone (<520 pmol/L)	272	854 (602–1189)	964 (680–1430)	748 (526–1022)^b^
PRA (1.5–5.7 μg/L/h)	239	0.2 (0.2–0.3)	0.2 (0.2–0.3)	0.2 (0.2–0.5)^a^
DRC (4.4–46 mU/L)	29	4.8 (2.3–7.9)	3.2 (1.9–5.4)	5.6 (4.9–8.7)^a^
ARR, serum aldosterone/PRA	238	3655 (2007–6732)	4208 (2554–7875)	2680 (1236–4364)^b^
ARR, serum aldosterone/DRC	29	220 (945–347)	263 (118–597)	145 (59–225)
24-h urine aldosterone (<40 nmol)	166	73 (50–106)	88 (61–124)	57 (42–85)^b^
Positive hypercortisolism screening test, *n* (%)	140	18 (6.5)	7 (8.6)	11 (26.8)^a^
Lowest plasma K^+^ (3.3–5.2 mmol/L)	268	3.0 ± 0.4	2.8 (2.6–3.1)	3.1 (2.9–3.3)^b^
Plasma creatinine (male 60–100 μmol/L, female 50–90 μmol/L)	276	76.0 ± 18.5	71 (61–83)	76 (68–89)^a^
Antihypertensive medication, DDD	270	3.5 (1.7–5.1)	3.3 (1.8–4.7)	4.0 (1.6–5.8)

Data are presented as number and percentage, mean ± s.d., or median (interquartile range). Letters ‘a’ and ’b’ indicate significant differences between APA and non-APA groups.

^a^*P* < 0.05; ^b^*P* < 0.001.

APA, aldosterone-producing adenoma; ARR, aldosterone–renin ratio; BMI, body mass index; BP, blood pressure; DDD, daily defined dose; DRC, direct renin concentration; PRA, plasma renin activity.

### Adrenalectomy outcomes according to histopathology

Adrenalectomy outcomes and various CYP11B2-positive histopathological findings in the APA and non-APA groups are shown in Table 2. As expected, after adrenalectomy the APA group had markedly higher rates of both biochemical and clinical cures (OR 13.2, 95% CI 3.9–45.5 and OR 12.9, 95% CI 6.1–27.3, respectively) with significantly higher plasma potassium, lower blood pressure, greater systolic blood pressure reduction, fewer antihypertensive medications, and greater reduction in the DDD of antihypertensive medications.

**Table 2:** Postoperative clinical characteristics and adrenal histopathology of patients with APA and non-APA.

Variable (reference range, unit)	Data available, *n*	All patients, *n* = 277	APA, *n* = 167	Non-APA, *n* = 110
**Postoperative variables**				
Systolic BP, mm Hg	226	131 (124–141)	130 (120–135)	140 (129–150)^b^
Systolic BP reduction, mm Hg	203	17 (6–30)	20 (9–40)	12 (4–25)^a^
Diastolic BP, mm Hg	226	80 (76–88)	80 (76–85)	83 (77–91)^a^
Diastolic BP reduction, mm Hg	203	10 (1–18)	10 (3–20)	9 (0–17)
Antihypertensive medication, DDD	273	1.1 (0.0–3.5)	0.57 (0.0–2.2)	2.5 (1.0–5)^b^
DDD reduction	265	1.3 (0.6–3.0)	2.0 (0.7–3.3)	0.8 (0.0–2.2)^b^
Plasma K^+^ (3.3–5.2 mmol/L)	266	4.0 (3.8–4.3)	4.1 (3.8–4.3)	3.9 (3.7–4.2)^b^
**Histopathology**				
CYP11B2-negative adenoma, *n* (%)	277	48 (17.3)	NA	48 (43.6)^b^
No adenoma, *n* (%)	277	62 (22.4)	NA	62 (56.4)^b^
Presence of APN, *n* (%)	277	40 (14.4)	13 (8.4)	27 (32.5)^b^
Presence of APM, *n* (%)	277	231 (83.4)	137 (82.0)	94 (85.5)
Number of APNs/APMs, *n*	277	3.0 (1.0–6.0)	2.0 (1.0–5.0)	4.0 (1.75–8.0)^b^
Presence of APDH, *n* (%)	277	28 (10.1)	15 (8.9)	13 (16.5)
Presence of any nonclassical feature, *n* (%)	277	243 (87.7)	139 (83.2)	104 (94.5)^a^
CYP11B2 score	277	NA	240.0 (165.0–270.0)	NA
CYP11B1 score	277	50.0 (10.0–100.0)	30.0 (10.0–70.0)	100.0 (26.3–163.8)^b^

Data are presented as number and percentage, mean ± s.d., or median (interquartile range). Letters ‘a’ and ’b’ indicate significant differences between APA and non-APA groups.

^a^*P* < 0.05; ^b^*P* < 0.001.

APA, aldosterone-producing adenoma; APDH, aldosterone-producing diffuse hyperplasia; APN, <7 mm CYP11B2-positive nodule; APM, aldosterone-producing micronodule; BP, blood pressure; DDD, daily defined dose; NA, not applicable.

The frequency of APN and number of APNs and APMs were higher in the non-APA group; however, APM presence was equally common in both groups. In the non-APA group, 48 patients (43.6%) had a CYP11B2-negative adenoma, while 62 (56.4%) had no adenoma present according to the re-evaluated H&E staining.

The total number of APNs and APMs correlated positively with patient age (*ρ* = 0.16, *P* < 0.009) and preoperative potassium level (*ρ* = 0.22, *P* < 0.001) and negatively with ARR (*ρ* = –0.18, *P* = 0.005) and the DDD of preoperative antihypertensive medication (*ρ* = –0.26, *P* < 0.001). There was no correlation with preoperative BP or BMI (data not shown). Five patients (1.8%) had no CYP11B2 positivity.

### Histopathology and adrenalectomy outcomes according to lateralization methods

All patients had either adrenal CT (94.6 %) or MRI (5.4%) performed, with 252 (91.0%) exhibiting an adrenal mass on the adrenalectomy side in imaging. CT- or MRI-based adrenalectomy was performed on 179 (64.6%) patients, whereas 43 (15.5%), 14 (5.4%), and 35 (12.6%) patients were operated based on lateralization in AVS, ^11^C-MTO-PET, and adrenal scintigraphy, respectively (Supplementary Table 1, see section on supplementary materials given at the end of this article). Six patients were excluded from the analysis, two due to unavailable CT results and four due to adrenalectomy despite normal/bilateral findings in both CT and adrenal scintigraphy.

Preoperative and postoperative characteristics and histopathological findings in patients operated based on anatomical imaging, pooled functional methods, and AVS, are presented in Table 3. The comparison of anatomical imaging with every different functional subtyping method is displayed in Supplementary Table 1. Supplementary Fig. 1 presents the adrenalectomy results according to anatomical and pooled functional subtyping methods. Adrenalectomy in the pooled functional method group of patients achieved complete or partial clinical cure more often than in the anatomical imaging group (88.8% vs 72.5%, *P* = 0.002), although there was no statistical difference between the biochemical cure rates, which were high overall. AVS-guided operations more likely resulted in complete or partial clinical cure than those based on CT/MRI (odds ratio (OR) 3.4, 95% CI 1.2–10.2) with a significantly larger decrease in systolic blood pressure (*P* = 0.02).

**Table 3:** Comparison of pre- and postoperative characteristics, histopathology and adrenalectomy outcomes between anatomical imaging with CT or MRI versus functional subtyping methods. Data for the AVS subgroup (part of functional method group) are also presented.

Variable (reference range, unit)	Anatomical imaging, *n* = 179	All functional methods, *n* = 92	AVS, *n* = 42
**Preoperative variables**			
Systolic BP, mm Hg	150 (135–170)	156 (141–165)	158 (145–170)
Diastolic BP, mm Hg	93 (84–100)	92 (85–100)	92 (86–96)
Lowest plasma K^+^, mmol/L	2.9 (2.7–3.2)	2.9 ± (2.7–3.1)	2.9 (2.6–3.0)^a^
Antihypertensive medication, DDD	3.1 (1.3–4.9)	4.0 (2.3–5.6)^a^	4.3 (6.7–6.4)^a^
Concordant CT/MRI lateralization	179 (100)	75 (81.5)	32 (74.4)
**Postoperative variables**			
Systolic BP reduction, mm Hg	13 (3–28)	20 (10–34)^a^	21 (14–35)^a^
Diastolic BP reduction, mm Hg	8 (−2 to 18)	11 (6–20)^a^	11 (5–17)
DDD reduction	1.3 (0.3–3.0)	1.9 (0.7–3.0)	1.8 (0.7–3.0)
Plasma K^+^, mmol/L	4.0 (3.8–4.2)	4.1 (3.8–4.4)^a^	4.1 (3.8–4.4)
**Histopathology**			
APA, *n* (%)	105 (58.7)	60 (65.2)	28 (65.1)
CYP11B2-negative adenoma	37 (20.7)	9 (9.8)^a^	5 (11.6)
No adenoma, *n* (%)	37 (20.7)	23 (25.0)	10 (23.3)
APN present, *n* (%)	27 (15.1)	11 (12.0)	6 (14.0)
Any nonclassical feature present, *n* (%)	160 (89.4)	77 (83.7)	34 (79.0)
CYP11B1 score	57.5 (18.8–101.3)	25.0 (6.0–87.5)^a^	20.0 (5.0–82.5)^a^

Data are presented as number and percentage, mean ± s.d., or median (interquartile range). ^a^indicates significant differences for pooled functional methods and AVS versus anatomical imaging.

^a^*P* < 0.05.

APA, aldosterone-producing adenoma; AVS, adrenal venous sampling; BP, blood pressure; CT, computer tomography; DDD, daily defined dose; MRI, magnetic resonance imaging.

There was no difference in the frequency of APAs or other CYP11B2 positive features between anatomical imaging and functional methods, although CYP11B2-negative adenomas were less common and CYP11B1 scores lower in the pooled functional method group.

In the AVS group, LI values were significantly higher in those with APA than those with non-APA (26.0 (13.3–95.7) vs 8.1 (5.0–19.4), *P* = 0.002). There was a positive correlation between LI values and CYP11B2 score (*ρ* = 0.33, *P* = 0.033).

### Comparison of patients with and without cure

Comparison of clinical characteristics and histopathological findings according to adrenalectomy outcomes is shown in Table 4. Older age, cardiovascular disease, higher antihypertensive medication doses, and a positive hypercortisolism screening test were associated with the absence of biochemical and clinical cure. The absence of clinical cure was additionally predicted by male sex, longer duration of hypertension, higher ARR, and higher 24-h urine aldosterone.

**Table 4:** Comparison of preoperative clinical characteristics and histopathological findings according to adrenalectomy outcomes.

Variable	Biochemical cure		Clinical cure	
	Present, *n* = 244	Absent, *n* = 25	Complete or partial, *n* = 203	Absent, *n* = 58
Sex, female/male	124/120	10/15	113/90	19/39^a^
Age, years	51.8 ± 11.4	58.7 ± 8.8^b^	50.7 ± 11.6	57.1 ± 10.8^b^
BMI, kg/m²	29.5 ± 5.4	29.6 ± 5.1	29.1 ± 5.5	30.2 ± 5.3
Hypertension duration, years	7.0 (2.0–15.0)	11.0 (7.0–14.0)	7.0 (2.0–13.0)	11.0 (7.0–20.0)^a^
Diabetes, *n* (%)	43 (17.8)	7 (28.0)	33 (16.4)	16 (27.6)
Cardiovascular disease, *n* (%)	16 (7.1)	8 (32.0)^b^	13 (6.5)	11 (19.0)^a^
Systolic BP, mm Hg	150 (139–165)	165 (140–175)	152 (140–167)	152 (140–170)
Diastolic BP, mm Hg	92 (85–100)	92 (86–100)	94 (85–100)	90 (82–100)
Antihypertensive medication, DDD	3.3 (1.6–5.0)	5.2 (3.5–6.5)^a^	3.3 (1.5–5.0)	4.3 (2.0–6.1)^a^
Lowest plasma K^+^ (3.3–5.2 mmol/L)	2.9 (2.7–3.2)	3.0 (2.8–3.3)	2.9 (2.7–3.1)	2.9 (2.8–3.3)
ARR, serum aldosterone/PRA (pmol/μg/h)	3793 (1983–6755)	2909 (2182–8673)	3818 (2181–7175)	2816 (1448–5978)^a^
24-h urine aldosterone (<40 nmol)	71.5 (49.8–105.3)	70.0 (44.0–113.0)	73.5 (54.8–106.5)	57.0 (40.0–86.0)^a^
Positive hypercortisolism screening test, *n* (%)	12 (4.9)	6 (24.0)^a^	9 (4.4)	9 (15.5)^a^
**Histopathology**				
APA, *n* (%)	157 (64.3)	3 (12.5)^b^	148 (72.9)	10 (17.2)^b^
CYP11B2-negative adenoma, *n* (%)	45 (18.4)	3 (12.5)	27 (13.3)	15 (25.9)^a^
No adenoma, *n* (%)	42 (17.2)	19 (79.2)^b^	28 (13.8)	33 (56.9)^b^
Presence of APN, *n* (%)	35 (14.3)	4 (16.7)	19 (9.4)	13 (22.4)^a^
Presence of APM, *n* (%)	204 (83.6)	21 (87.5)	168 (82.8)	50 (86.2)
Presence of APDH, *n* (%)	24 (9.8)	2 (8.3)	25 (12.3)	3 (5.2)
CYP11B1 score	50.0 (10.0–100.0)	75.0 (15.0–210.0)	40.0 (12.0–134.0)	72.5 (45.0–88.0)

Data are presented as number and percentage, mean ± s.d., or median (interquartile range). Letters ‘a’ and ‘b’ indicate significant differences between (i) absent versus present biochemical cure and (ii) absent versus complete or partial clinical cure.

^a^*P* < 0.05; ^b^*P* < 0.001.

APA, aldosterone-producing adenoma; APDH, aldosterone-producing diffuse hyperplasia; APN, <7 mm CYP11B2-positive nodule; APM, aldosterone-producing micronodule; ARR, aldosterone–renin ratio; BMI, body mass index; BP, blood pressure; DDD, daily defined dose; PRA, plasma renin activity.

APNs were more 2.4 times more frequent in patients with absent clinical cure than those with complete or partial cure (22.4% vs 9.4 %, *P* = 0.012) (Table 4). However, the prevalence of APMs and APDH was similar between those with absent vs partial or complete cure (86.2% vs 82.6 %, *P* = 0.69 and 5.2% vs 12.3%, *P* = 0.15, respectively). Of the 48 patients with a CYP11B2-negative adenoma, 93.8% had biochemical cure, while biochemical cure was achieved in 68.9% of the 61 patients with no adenoma (*P* = 0.001). There was no significant difference in achieving partial or complete clinical cure between those with CYP11B2-negative adenoma compared to those with no adenoma (64.3% vs 45.9%, *P* = 0.074).

### Evaluation of cortisol cosecretion and CYP11B1 immunostaining

A positive screening test for hypercortisolism was more common in patients with non-APA (*P* = 0.035) (Table 1), in patients operated according to anatomical imaging (*P* = 0.032) (Table 3), and in those with absent biochemical or clinical cure (*P* = 0.002 and *P* = 0.001, respectively) (Table 4). In histopathological examination, CY11B1 score was significantly lower in patients with APA versus non-APA (*P* < 0.001) (Table 2), and in the functional method group than in the anatomical imaging group (*P* = 0.016) (Table 3). Conversely, CYP11B1 score was higher in those with a CYP11B2-negative adenoma than in the remaining patients (120.0 (10.0–90.0) vs 30.0 (70.0–180.0), *P* < 0.001). There was a positive correlation between CYP11B1 score and serum cortisol value in dexamethasone suppression test (*ρ* = 0.35, *P* = 0.005). However, CYP11B1 score did not differ among patients who achieved biochemical or clinical cure and those who did not when considering all patients.

### Determinants of clinical cure after adrenalectomy

A logistic regression analysis was performed to evaluate the effect of patient sex, age, cardiovascular disease, DDD of antihypertensive medication, hypercortisolism screening test positivity, ARR, and presence of APA and APN on achieving partial or clinical cure of PA (Table 5). As hypertension duration highly correlated with patient age (*r* = 0.56, *P* < 0.001) and 24-h urine aldosterone correlated with ARR (*ρ* = 0.23, *P* < 0.001), only age and ARR were chosen for the model. The model was statistically significant, *χ* ^2^ 42.029, *P* < 0.001, explaining 50.7% (Nagelkerke *R^2^*) of the variance in clinical cure of PA and correctly classifying 83.8% of cases. Less intensive antihypertensive medication, use of functional subtyping method, and the finding of an APA in CYP11B2 staining emerged as independent predictors of achieving a clinical cure.

**Table 5:** Binary logistic regression model for the determinants of complete or partial clinical cure.

Variable	Coefficient	Odds ratio	95% CI	*P*
Age	−0.04	0.96	0.91–1.02	0.207
Male sex	−0.31	0.74	0.19–2.87	0.658
Presence of cardiovascular disease	−0.31	0.73	0.13–4.10	0.721
DDD of antihypertensive medication	−0.28	0.75	0.59–0.96	**0.024**
Preoperative ARR (serum aldosterone/PRA)	0.00	1.0	1.00–1.00	0.781
Positive hypercortisolism screening test	−1.44	0.24	0.05–1.09	0.064
Functional subtyping method was applied in diagnostics	2.60	13.48	1.85–98.40	**0.010**
Presence of APA	1.95	7.00	1.86–26.36	**0.004**
Presence of APN	−0.55	0.58	0.07–5.09	0.622
Constant	3.42	30.43		**0.045**

Values in bold indicate statistical significance.

APA, aldosterone-producing adenoma; APN, <7 mm CYP11B2-positive nodule; ARR, aldosterone–renin ratio; DDD, daily defined dose; PRA, plasma renin activity.

## Discussion

Deciding when to proceed with surgical treatment and assessing the surgical outcome are crucial steps in mitigating the increased risk of metabolic and cardiovascular complications associated with PA (1, 2, 3, 4, 5, 6, 7). Therefore, our objective was to evaluate the value of immunohistochemical analyses of CYP11B2 and CYP11B1 according to adrenalectomy outcomes as well as subtyping method applied. Using CYP11B2 immunohistochemistry appeared essential as it improved the original histopathological H&E-based diagnosis in one third of the subjects. The finding of CYP11B2 positive adenoma strongly associated with achieving cure from PA. However, if APA was presented together with APNs, the possibility of later clinical recurrence in the contralateral gland should be considered even among those with initial improvement in clinical and biochemical profile. We found that adrenalectomy guided by functional subtyping achieved greater rates of clinical cure than operations guided by anatomical subtyping, even though there was no difference in the rate of CYP11B2 positive findings according to the subtyping method.

The presence of APN was associated with less favorable surgical outcome, suggesting probable asymmetric bilateral disease (25), and the possibility of later recurrence of autonomous PA in the contralateral gland. Importantly, almost every tenth patient with APA in our study also had one or multiple APNs, highlighting the importance of immunohistochemical examination. The prevalence of APMs was high in all patients in this study, and in contrast to the findings of Wu *et al.*, Meyer *et al.*, and Lin *et al.* (10, 12, 13), the presence of APMs or APDH was not associated with adrenalectomy outcome. Aldosterone-producing cell clusters, which have been renamed as APMs (8), accumulate with age in normal adrenals of non-hypertensive persons (26). Accordingly, in our study, the number of APNs and APMs significantly correlated with patient age. Altogether, the prevalence of all nonclassical features (APN, APM, and APDH) was high (87.7%) in the study population. The clinical significance of the histopathological findings (8) will be clarified in long-term follow-up studies in the future.

Interestingly, a higher rate of biochemical cure was achieved in patients with a CYP11B2-negative adenoma compared to those with no adenoma. This might partially be explained by the higher CYP11B1 scores in adrenal samples with CYP11B2-negative adenomas, implicating possible cortisol cosecretion (27, 28). Another possibility is that histopathological methods did not reveal every source of autonomous aldosterone secretion. According to the Monticone *et al.*, CYP11B2 expression is not captured in some cases with immunohistochemistry despite verified clinical and biochemical cure (29).

Those who presented with a non-APA exhibited increased rates of comorbidity including cardiovascular disease, the prevalence of which was 3.7 times higher than in those with APA. This high-risk profile was present despite a milder biochemical and clinical phenotype of aldosteronism, as described in literature (30). Although this finding might be partially explained by the older age and longer PA duration of patients with non-APA, bilateral disease is clearly not without consequence. Our findings highlight the need to identify patients with high cardiovascular risk among patients with non-APA, as preliminary evidence suggests that adrenalectomy may be beneficial even for some selected patients with bilateral PA (31).

Adrenalectomy guided by functional subtyping achieved greater rates of blood pressure and antihypertensive medication reductions and increases in plasma potassium than operations guided by anatomical subtyping, but there was no difference in the prevalence of APAs or other CYP11B2 positive findings (Table 3, Supplementary Table 1). This finding reflects the differences in patient groups undergoing surgery based on anatomical imaging and functional methods. Functional methods are directed at detecting lateralization of aldosterone production. It is well established that unilateral aldosteronism is partly caused by CYP11B2-positive nodules and micronodules, which remain unrecognized by anatomical imaging (25). Therefore, patients operated according to functional methods may have unilateral disease even in the absence of an APA. Patients in anatomical imaging group were, by definition, identified with a visible nodule in CT or MRI. Every fifth of these cases turned out to be CYP11B2-negative adenomas, while this was the case for only every tenth patient in the pooled functional method group. Additionally, AVS results reflected histopathological findings in the sense that CYP11B2 scores calculated from APAs correlated with LI values, indicating an association between immunohistochemical findings and the degree of aldosterone hypersecretion.

Additionally, the pooled functional method group exhibited lower CYP11B1 scores and less frequent positive hypercortisolism screening results than the anatomical imaging group. In a subset of APAs, cortisol cosecretion (27, 28) may interfere with interpretation of AVS results, when routine cortisol measurement is used for calculation of selectivity and lateralization indexes (32). Theoretically, functional subtyping methods may miss some of the unilateral cases with a cortisol-secreting APA (27, 28), but the risk of inadvertent operation still remains higher in adrenalectomy based on anatomical imaging.

The use of functional subtyping was independently predictive of achieving clinical cure in regression analysis. In fact, the risk of absent clinical cure was 2.5-fold higher after anatomical imaging despite a milder clinical phenotype. Our results are consistent with the meta-analysis of Yan *et al.* (33) on the superiority of AVS compared to CT/MRI. In line with recent prospective trials (34, 35), those who underwent a ^11^C-MTO-PET-guided operation achieved comparable biochemical cure as AVS-guided operation but the small numbers of subjects must be considered in our subgroup analyses. As expected, adrenal scintigraphy detected only large APAs, and this method has been abandoned due to its low sensitivity in PA subtyping (17).

The strength of our study is the histopathological examination retrospectively performed on all adrenal samples, with findings evaluated in relation to clinical and biochemical parameters and the achieved cure of PA. To our knowledge, this is the largest study that has performed CYP11B2 staining on adrenalectomized patients with PA. Although our study was designed and executed before the publication of the HISTALDO consensus, our histopathological analysis was generally consistent with this consensus except for the small difference in the definition APA, which in our study was a solitary CYP11B2-positive neoplasm ≥7 mm in diameter, and accordingly the definition of APN, which in our study was a round CYP11B2-positive nodule <7 mm in diameter whereas the cutoff in HISTALDO study was 10 mm (8). The presence of APA according to this definition correlated well with the achieved cure of PA.

Our study has some limitations. The retrospective data recorded in the patient registry did not permit following the PASO criteria strictly. Additionally, the limited numbers of patients who were operated based on the three functional methods limited the possibility to evaluate these methods separately. A sampling error is possible in histopathological studies and could possibly explain the finding of five CYP11B2-negative samples. However, we studied all available diagnostic H&E sections per adrenal gland and selected the most representative whole sections for immunohistochemistry.

To conclude, immunohistochemical CYP11B2 staining changed the histopathological diagnosis in a third of patients and commonly revealed nonclassical histology even in patients found to have an APA and presenting with a clinical cure. Although no current method of assessing the lateralization of aldosterone overproduction is without limitations, we found higher clinical cure rates and lower prevalence of CYP11B2 negative adenomas among those who underwent functional lateralization studies. According to our findings, the finding of an APA in histopathological examination represents an independent predictor of achieving clinical cure after adrenalectomy. Whether patients with non-APA have persistently increased risk for comorbidities such as cardiovascular disease remains to be established. Thus, immunohistochemical CYP11B2 analysis improves the evaluation of surgical outcome and should be part of clinical assessment of PA. Future studies with long-term follow-up are warranted to assess the cure of PA in relation to CYP11B2 staining.

## Supplementary materials

Supplementary Material

## Declaration of interest

The authors have nothing to disclose. Niina Matikainen is an Editorial Board member of *Endocrine Connections*. Niina Matikainen was not involved in the review or editorial process for this paper, on which she is listed as an author.

## Funding

This work was supported by a research grant from the Helsinki University Hospital Competitive State Research Financing (VTR TYH2020402 and TYH2022311, NM), the Koskelo Foundation (NM), the Finnish Foundation for Cardiovascular research (IP), and the Sigrid Jusélius Foundation (IP). Open access funded by Helsinki University Library.
