# Supplementary material for: Clinical significance of CYP11B2 immunostaining in unilateral primary aldosteronism
Source: Endocr Connect. 2024 Jan 12;13(2):e230344. doi: 10.1530/EC-23-0344 (PMC10831582; doi:10.1530/EC-23-0344)

Clinical significance of CYP11B2 immunostaining in unilateral primary aldosteronism

Marianna Viukari et al.

Supplemental methods

Preoperatively or postoperatively, 75 patients lacked blood pressure data and 9 patients lacked data on antihypertensive medication doses, hence clinical cure was classified according to either reduction of antihypertensive medication or blood pressure alone. Only 74 patients had postoperative aldosterone and renin measured, and if available, the measurements were mostly performed because of poor clinical outcome or persistently low potassium, creating bias. Consequently, we used modified PASO criteria to define biochemical cure based on achieving normokalaemia without potassium supplements or MRA medication. Therefore, the outcome of biochemical cure was categorized as present or absent. After adrenalectomy, 21 patients were not considered biochemically or clinically cured due to the need to restart MRA medication. Biochemical and clinical cure could not be determined in 8 and 16 cases, respectively.

Supplemental results

Adrenalectomy results according to concordance between anatomical imaging and functional subtyping

In the AVS subgroup, 11 (25.6%) patients had discrepancy between preoperative lateralization in AVS and CT/MRI (**Table 3**). However, no statistical difference in the biochemical or clinical cure rates when compared with patients with concordant CT/MRI and AVS lateralization (data not shown) was observed. The same applies to the 5 patients with discordant CT/MRI findings in the ^{11}C-MTO-PET subgroup. In the adrenal scintigraphy subgroup, every patient with biochemical or clinical cure had concordant CT/MRI findings, whereas the single patient with a discordant CT

finding was had non-APA and had neither biochemical nor clinical cure. Additionally, in two cases, adrenal scintigraphy showed no lateralization and adrenalectomy was performed according to CT, resulting in both biochemical and clinical cure. The findings of the subgroup who underwent both AVS and ^{11}C-MTO-PET have been reported previously[25].

Adrenalectomy outcome of young patients

Of the 20 patients aged ≤35 years, 18 (90%) fulfilled the definition of severe PA eligible for adrenal surgery based on anatomical subtyping[12] and 16 (80%) underwent anatomical imaging only. One patient underwent AVS and 2 had adrenal scintigraphy; all 3 had findings concordant with CT lateralization. One patient (5%) did not have lateralization in CT and was operated according to lateralization in ^{11}C-MTO-PET, resulting in complete biochemical and clinical cure. Overall, among the younger patients, 100% achieved biochemical and 93.8% complete or partial clinical cure. Among patients aged >35 years, the corresponding cure rates were 88.7% and 70.2%, although the differences were not statistically significant when compared with younger patients. APA was found in 19/20 of the young patients (95%), which was a significantly larger proportion ($p<0.001$) when compared with 148/257 (57.6%) older patients (data not shown).

Supplemental Table. Comparison of pre- and postoperative characteristics, histopathology, and adrenalectomy outcomes between anatomical imaging with CT or MRI versus functional subtyping methods.

Variable (reference range, unit)		CT/MRI n=179	AVS n=43	^{11}C-MTO-PET n=14	Adrenal scintigraphy n=35
**Preoperative variables**					
Systolic BP, mmHg		150 [135–170]	158 [145–170]	158 [137–164]	150 [136–160]
Diastolic BP, mmHg		93 [84–100]	92 [86–96]	94 [80–100]	92 [88–100]
Lowest plasma K^+, mmol/l		2.9 [2.7–3.2]	2.9 [2.6–3.0]*	2.8 [2.7–3.2]	3.0 [2.9–3.2]
Antihypertensive medication, DDD		3.1 [1.3–4.9]	4.3 [6.7–6.4]*	4.3 [1.6–5.1]	3.7 [2.3–5.0]
Concordant CT/MRI lateralization		179 (100)	32 (74.4)	9 (64.3)	34 (97.1)
**Postoperative variables**					
Systolic BP reduction, mmHg		13 [3–28]	21 [14–35]*	15 [1–34]	20 [10–35]
Diastolic BP reduction, mmHg		8 [-2–18]	11 [5–17]	13 [1–20]	12 [8–22]
DDD reduction		1.3 [0.3–3.0]	1.8 [0.7–3.0]	1.3 [0.7–3.4]	2.0 [0.7–2.7]
Plasma K^+, mmol/l		4.0 [3.8–4.2]	4.1 [3.8–4.4]	3.9 [3.7–4.4]	4.2 [4.0–4.3]*
**Histopathology**					
APA, n (%)		105 (58.7)	28 (65.1)	8 (51.7)	24 (68.6)
Presence of any non-classical feature, n (%)		160 (89.4)	34 (79.0)	14 (100)	29 (82.3)
**Adrenalectomy outcomes**					
Biochemical cure	Complete, n (%)	155 (89.6)	40 (95.2)	14 (100)	31 (91.2)
	Absent, n (%)	18 (10.4)	2 (4.8)	0 (0)	3 (8.8)
Clinical cure	Complete, n (%)	65 (38.9)	9 (22.5)	4 (28.6)	12 (34.3)
	Partial, n (%)	56 (33.5)	27 (67.5)	9 (64.3)	18 (51.4)
	Absent, n (%)	46 (27.5)	4 (10.0)*	1 (7.1)	5 (14.3)

Number and percentage, mean ± standard deviation, or median [interquartile range]; APA, aldosterone-producing adenoma; AVS, adrenal venous sampling; BP, blood pressure; ^{11}C-MTO-PET; metomidate positron emission tomography; CT, computer tomography; DDD, daily defined dose; MRI, magnetic resonance image; n, number. Asterisks indicate significant differences for each functional method (AVS/^{11}C-MTO-PET/adrenal scintigraphy) versus CT/MRI; *P<0.05; ** P<0.001.

Supplemental Figure. Comparison of biochemical and clinical cure according to subtyping method.

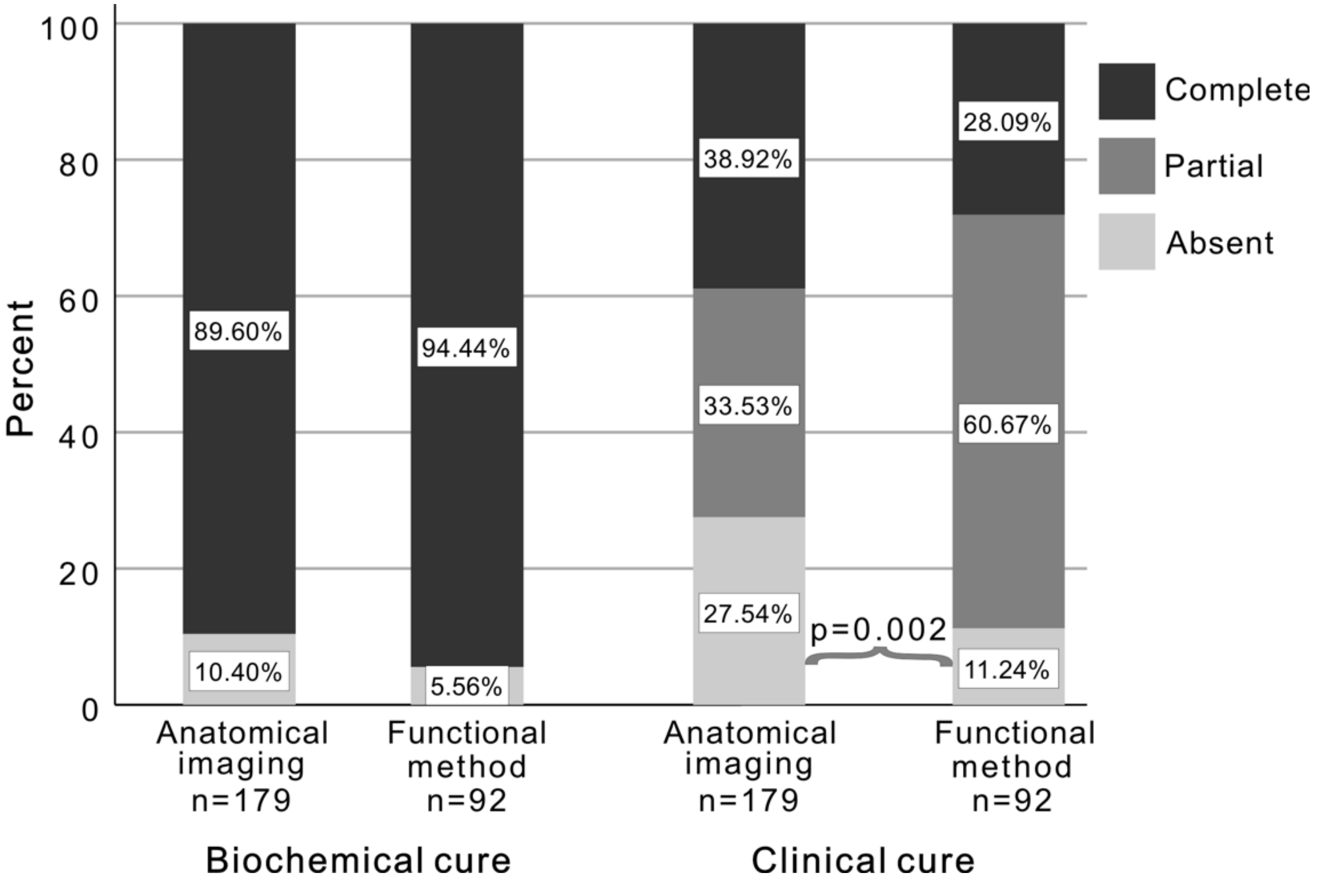

Supplement: Supplementary Material [file supplementary_material.pdf]
